
JOURNAL INFORMATION
==============================
NLM Title Abbreviation: Innov Aging
Journal ID: Innov Aging
NLM Journal ID: 101703706
Journal ID: innovateage

Innovation in Aging

EISSN: 2399-5300
Publisher: Oxford University Press

ARTICLE INFORMATION
==============================
PMCID: PMC6846214
DOI: 10.1093/geroni/igz038.995
Article ID: igz038.995
Article version: 1
Subjects: Session 1330 (Poster); Depression, Anxiety, and Mental Health

SELF-REPORTED VISION IMPAIRMENT, VISION CORRECTION, AND DEPRESSION AMONG MIDDLE-AGED AND OLDER CHINESE

Xu Beibei 1
Cao Gui-Ying 2
Yao Shan-Shan 2
Chen zi-shuo 2
Huang Zi-Ting 2
1 Peking University Medical Informatics Center , Beijing, China
2 Department of Epidemiology and bio-statistics School of Public Health , Peking University, Beijing, China
Publication date: 2019 Nov
Electronic publication date: 2019 Nov 8
Volume: 3
Issue: Suppl 1
Issue title: Program Abstracts from the GSA 2019 Annual Scientific Meeting “Strength in Age—Harnessing the Power of Networks”
First page: S268
Last page: S268
Copyright: © The Author(s) 2019. Published by Oxford University Press on behalf of The Gerontological Society of America.
Copyright year: 2019
License: This is an Open Access article distributed under the terms of the Creative Commons Attribution License (http://creativecommons.org/licenses/by/4.0/), which permits unrestricted reuse, distribution, and reproduction in any medium, provided the original work is properly cited.
License URL: https://creativecommons.org/licenses/by/4.0/

Page count: 1

==============================
### SELF-REPORTED VISION IMPAIRMENT, VISION CORRECTION, AND DEPRESSION AMONG MIDDLE-AGED AND OLDER CHINESE

Beibei Xu,[1] Gui-Ying Cao,[2] Shan-Shan Yao,[2] zi-shuo Chen,[2] and Zi-Ting Huang[2], *1. Peking University Medical Informatics Center, Beijing, China, 2. Department of Epidemiology and bio-statistics School of Public Health, Peking University, Beijing, China*

This study utilized data from the China Health and Retirement Longitudinal Study (CHARLS) to investigate the associations between vision impairment (VI), vision correction (VC) and depression among middle-aged and older Chinese. Near or distance vision and VC were reported via questionnaires. For this study, participants were divided into four categories: no VI, distance vision impairment (DVI), near vision impairment (NVI), or both distance and near vision impairment (DNVI). Participants were also divided into four groups: no VI with VC (VI(-)/VC(+)), no VI without VC (VI(-)/VC(-)), VI with VC (VI(+)/VC(+)) and VI without VC (VI(+)/VC(-)). Depression was evaluated by Center for Epidemiological Studies Depression Scale (CES-D-10). Of 16,159 individuals aged 45 years and older included, the mean (SE) age was 58.5 (0.1) years and 52% of participants were women. Compared with no VI, DVI only (OR=1.92; 95% CI: 1.69, 2.19), NVI only (OR=1.34; 95% CI: 1.17, 1.53) and DNVI (OR=2.23; 95% CI: 1.86, 2.66) were associated with higher odds of depression at baseline after full adjustment with covariates. Only DNVI was significantly associated with higher odds of onset of depression two (OR=1.41; 95% CI: 1.12, 1.78) and four (OR=1.30; 95% CI: 1.02, 1.66) years later after full adjustment. Compared with VI(-)/VC(+), VI(-)/VC(-) (OR=1.36; 95% CI: 1.03, 1.81) and VI(+)/VC(-) (OR=1.55; 95% CI: 1.15, 2.10) were associated with higher odds of onset of depression four years later after full adjustment. VC might be a protective factor for preventing depression among those middle-aged and older individuals with VI or potential vision decline.
